# Association Between Air Pollution and Childhood Asthma: A Systematic Review of Recent Evidence

**DOI:** 10.3390/arm94030031

**Published:** 2026-05-12

**Authors:** Maria Kyrmanidou, Ioannis Smaraidos, Asterios Kampouras

**Affiliations:** 1 4th Pediatric Department, Aristotle University of Thessaloniki, 54124 Thessaloniki, Greece; kirmanidoumaria13@yahoo.gr; 2 Department of Economics, Directorate of Academics, Hellenic Military Academy of Combat Support Officers, 54636 Thessaloniki, Greece; smarelos@gmail.com

**Keywords:** air pollution, childhood asthma, PM_2.5_, PM_10_, nitrogen dioxide, pediatric respiratory health

## Abstract

**Highlights:**

**What are the main findings?**

- Long-term exposure to PM, PM and NO is consistently associated with a 15–30% increase in childhood asthma risk per 10 μg/m^3^ across large international and European cohort studies.
- Early-life exposure (prenatal and early childhood) represents a critical window of vulnerability, with stronger effects on asthma development, lung function decline, and exacerbations.

**What are the implications of the main findings?**

- Adverse respiratory effects occur even below current regulatory thresholds, supporting the need for stricter air-quality standards.
- Targeted urban interventions and improved longitudinal monitoring are essential to reduce the burden of childhood asthma, particularly in high-risk European populations.

**Abstract:**

Background: Air pollution is a major environmental determinant of respiratory health and a significant contributor to the global burden of childhood asthma. Although several recent narrative and systematic reviews have examined environmental triggers of asthma, highlighting air pollution as a consistent risk factor across diverse populations and study designs, recent epidemiological evidence—including multicenter cohort studies and region-specific analyses from Europe and Greece—has not been systematically synthesized. Objective: To systematically review recent epidemiological evidence (2000–2025) on the association between ambient air pollution and childhood asthma incidence and exacerbations, with emphasis on European and Greek populations. Methods: Following PRISMA guidelines, we systematically reviewed observational studies published between 2000 and 2025 in PubMed, Scopus, Web of Science, BMC, and Google Scholar. Studies evaluating quantitative exposure to PM_2.5_, PM_10_, NO_2_, O_3_, or SO_2_ and asthma incidence, prevalence, or exacerbations in children (≤18 years) were included. Evidence was synthesized by pollutant type, exposure window, geographic region, and study design. Results: Twenty-four studies involving more than 3.5 million children were included. Consistent associations were observed across international and European cohorts between long-term exposure to PM_2.5_, PM_10_, and NO_2_ and increased asthma incidence. Risk estimates typically ranged from 15% to 30% increases in asthma incidence per 10 μg/m^3^ increase in long-term exposure to PM_2.5_ or NO_2_, as reported across multiple cohort analyses. Early-life exposure showed the strongest effects on asthma development and lung function decline. European and Greek studies demonstrated comparable trends, highlighting increased hospitalizations and symptom burden in urban populations despite pollutant concentrations often below current regulatory thresholds. Short-term pollution peaks were additionally associated with increased asthma exacerbations and hospital admissions, particularly during seasonal episodes of elevated particulate matter and ozone concentrations. Conclusions: This review provides an updated synthesis of 21st-century evidence demonstrating that ambient air pollution is a major and modifiable determinant of childhood asthma. The consistency of findings across regions, combined with limited longitudinal evidence from Greece, highlight the importance of improved air-quality management and continued public-health efforts to reduce exposure and the need for enhanced epidemiological monitoring.

## 1. Introduction

Childhood asthma is one of the most prevalent chronic respiratory diseases worldwide, affecting millions of children and representing a leading cause of emergency visits, hospitalizations, and school absenteeism [1,2]. Its increasing prevalence—particularly in urbanized and industrialized regions—suggests an important contribution of environmental determinants beyond genetic susceptibility [3,4]. Global estimates indicate that environmental risk factors, including air pollution, contribute substantially to the burden of asthma and other respiratory diseases in children.

Ambient air pollution is widely recognized as a major environmental risk factor for global morbidity and mortality [5,6]. The World Health Organization updated its global air quality guidelines in 2021, recommending lower exposure limits for pollutants such as PM_2.5_ and NO_2_, reflecting evidence that adverse health effects occur even at relatively low concentrations [3]. Similarly, the Global Initiative for Asthma identifies outdoor air pollution as a key modifiable risk factor influencing both asthma development and exacerbations [7].

Ambient pollutants—including PM_2.5_, PM_10_, NO_2_, SO_2_, and O_3_—can induce oxidative stress, airway inflammation, epithelial injury, and immune dysregulation [4,6,8,9]. Traffic-related pollutants, particularly PM_2.5_ and NO_2_, are strongly associated with urban exposure gradients and adverse respiratory outcomes in children, including increased respiratory infections and asthma-related morbidity in urban settings [10,11,12].

Despite regulatory efforts, pollutant levels in many European cities remain above recommended thresholds [13], and adverse effects have been reported even below current limits. However, although numerous studies have investigated the association between air pollution and asthma, many earlier reviews predate recent large multicenter cohort studies and updated European data. In addition, region-specific evidence from Southern Europe, including Greece, remains incompletely synthesized.

Therefore, this systematic review aims to evaluate epidemiological evidence from 2000 to 2025 on the association between ambient air pollution and childhood asthma, with particular emphasis on European and Greek populations.

## 2. Materials and Methods

This systematic review was conducted in accordance with the PRISMA guidelines [14]. A comprehensive literature search was performed in PubMed, Scopus, Web of Science, BMC, and Google Scholar for studies published between January 2000 and March 2025. Google Scholar was additionally used to identify potentially relevant studies not indexed in traditional databases. No geographic restrictions were applied. The review protocol was not prospectively registered; however, all stages of the review process were conducted in accordance with PRISMA recommendations to ensure methodological transparency.

Search terms combined air-pollution exposures with childhood-asthma outcomes and geographic identifiers when relevant. Only peer-reviewed articles published in English were included. The last search was conducted on 15 March 2025.

**Figure 1:**
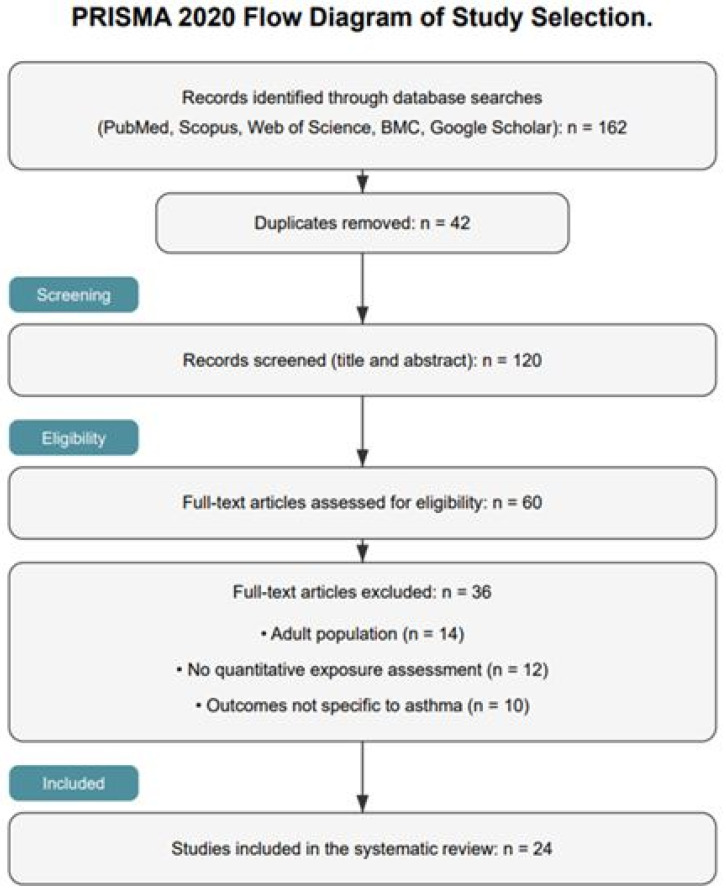
PRISMA flow diagram illustrating the process of study identification, screening, eligibility assessment, and inclusion in the systematic review.

Study screening followed a two-stage process performed independently by two reviewers based on predefined inclusion and exclusion criteria. Following title and abstract screening, a substantial proportion of records were excluded due to duplication or lack of relevance, and the remaining articles were assessed for full-text eligibility based on predefined inclusion criteria. Following title and abstract screening, 120 records were excluded due to duplication or irrelevance, and 60 articles were assessed for full-text eligibility based on predefined inclusion criteria. Of these, 36 articles were excluded due to lack of quantitative exposure assessment, non-specific asthma outcomes, or ineligible populations. Disagreements were resolved by consensus with a third reviewer. A total of 24 studies were included in the final analysis. The study-selection process is summarized in Figure 1.

### 2.1. Eligibility Criteria

Inclusion Criteria

- Observational epidemiological studies;
- Children ≤ 18 years;
- Quantitative exposure assessment (PM_2.5_, PM_10_, NO_2_, O_3_, SO_2_);
- Asthma incidence, prevalence, or exacerbation outcomes.

Although children were defined as individuals ≤ 18 years, several of the included studies further stratified participants into early childhood, school-age children, and adolescents when analyzing respiratory outcomes.

Exclusion Criteria

- Adult-only populations;
- Experimental or toxicological studies;
- Lack of quantitative exposure metrics;
- Outcomes not specific to asthma.

### 2.2. Quality Assessment

Study quality was assessed using the AXIS tool for cross-sectional studies [15] and the Newcastle–Ottawa Scale for cohort and case–control studies [16]. These tools have been widely used in epidemiological systematic reviews.

## 3. Results

### 3.1. Study Selection

The literature search identified 162 records. After duplicate removal and screening, 24 studies met inclusion criteria.

### 3.2. Characteristics of Included Studies

The included studies encompassed more than 3.5 million children across North America, Europe, and Asia. Approximately half of the included studies were prospective cohort studies, while the remainder consisted of cross-sectional and time-series analyses. Sample sizes ranged from fewer than 1000 participants in localized studies to over 300,000 children in large multicenter cohorts. Several studies specifically focused on early childhood populations (0–5 years), whereas others included school-age children and adolescents, allowing partial age-stratified analyses of respiratory outcomes. Exposure assessment was based on monitoring stations, land-use regression models, or satellite-derived estimates [3,10,16,17]. In most studies, long-term exposure referred to annual or multi-year average pollutant concentrations, whereas short-term exposure reflected daily or weekly pollution fluctuations associated with acute respiratory outcomes.

Table 1 summarizes the main methodological characteristics and key findings of representative studies included in the review, including study design, population characteristics, pollutants examined, and reported respiratory outcomes.

### 3.3. International Evidence on Air Pollution and Childhood Asthma

Large multicenter cohort studies consistently demonstrated associations between long-term exposure to PM_2.5_, PM_10_, and NO_2_ and increased asthma incidence [8,10,12,16,18]. Risk estimates ranged from approximately 15% to 30% per 10 μg/m^3^ increase in pollutant concentration [8,10,18]. Early-life exposure showed the strongest effects on asthma development and lung function decline, with stronger associations reported in early childhood compared with adolescence. Comparable effect sizes were observed across different geographic regions, including North America, Europe, and Asia, supporting the consistency of the association across diverse environmental and demographic contexts [9,10,12,16,18].

Short-term pollution peaks were associated with higher rates of emergency department visits and hospital admissions for asthma [4,8,18,23]. Ozone exposure was also linked to asthma morbidity in children [24].

Improved air quality has been associated with measurable improvements in lung development, supporting the reversibility of pollution-related effects [18,19].

Meteorological factors were reported to modify pollutant concentrations and related health outcomes [20,22]. Seasonal ozone peaks were linked to increased asthma exacerbations during warmer months [24].

Evidence regarding SO_2_ exposure and childhood asthma was limited and heterogeneous across the included studies, with some analyses suggesting weak or non-significant associations, preventing robust conclusions regarding its independent effect [8,9]. This inconsistency may reflect lower exposure variability, differences in measurement methods, or confounding with co-pollutants in urban environments.

### 3.4. European and Greek Evidence

European multicenter initiatives such as ESCAPE, GA^2^LEN, PIAMA, and BREATHE consistently reported increased asthma prevalence, impaired lung growth, and reduced lung function associated with traffic-related pollution [21,25,26,27]. These findings were supported by large cohort and cross-sectional studies across Europe demonstrating measurable respiratory effects even at pollutant concentrations below current EU regulatory limits [18,20,22].

Several epidemiological studies have quantified the magnitude of risk associated with major pollutants. Long-term exposure to PM_2.5_, PM_10_, and NO_2_ has been associated with approximately 15–30% increases in childhood asthma risk per 10 μg/m^3^ increase across multiple cohort analyses [8,10,18] (see Table 2). Seasonal ozone exposure has been linked to increased asthma exacerbations and hospitalizations in warmer months [24], with risk increases typically in the range of 10–15% depending on exposure intensity.

Greek studies, including ISAAC II and time-series analyses by Samoli et al. and Katsouyanni et al., reported higher asthma prevalence, increased hospital admissions, and reduced lung function among children living in polluted urban areas [20,22]. Although evidence from Greece remains relatively limited compared with larger international datasets Thessaloniki and Athens were repeatedly identified as high-risk urban environments, reflecting the combined effects of traffic emissions, urban density, and seasonal pollution episodes [20].

Overall, European and Greek evidence shows consistent associations between particulate matter, nitrogen dioxide, and childhood asthma outcomes, with effect sizes comparable to those reported in international studies and meta-analyses [1,2,3,10,16,18,25] (see Table 3).

## 4. Discussion

This systematic review synthesizes approximately 25 years of international epidemiological evidence (2000–2025) examining the relationship between ambient air pollution and childhood asthma. The findings demonstrate consistent associations between long-term exposure to particulate matter (PM_2.5_ and PM_10_) and nitrogen dioxide (NO_2_) and increased asthma incidence, as well as short-term associations with exacerbations and hospital admissions. A formal meta-analysis was not performed due to substantial heterogeneity in study design, exposure assessment, and outcome definitions across the included studies.

These findings are consistent with previous large-scale systematic reviews and meta-analyses, which have identified traffic-related air pollution as a major contributor to childhood asthma development. In particular, meta-analytic evidence by Orellano et al. and Bowatte et al. confirms significant associations between traffic-related pollutants and both asthma incidence and exacerbations in pediatric populations [29,30]. These studies report comparable effect estimates and reinforce the role of nitrogen dioxide and fine particulate matter as key environmental risk factors. The consistency of findings across independent populations and study designs strengthens the evidence supporting a likely causal relationship.

From a mechanistic perspective, experimental and clinical research provides strong biological plausibility for these associations. Air pollutants induce oxidative stress, airway inflammation, epithelial injury, and immune dysregulation. Exposure to fine and ultrafine particles has been shown to penetrate deep into the respiratory tract and trigger systemic inflammatory responses, while traffic-related pollutants contribute to airway hyperresponsiveness and allergic sensitization. Evidence from mechanistic studies further supports the biological plausibility of air pollution–induced asthma development [31].

The review highlights early-life exposure—including prenatal and early childhood periods—as a critical window of vulnerability. This observation is consistent with developmental models of respiratory disease and supported by the broader evidence base on air pollution and lung development, indicating that early environmental exposures can result in long-term impairment of respiratory function [32].

Importantly, adverse respiratory effects were observed even at pollutant concentrations below current regulatory thresholds. This finding aligns with evidence from international health assessments, including reports from the World Health Organization, which emphasize that no safe exposure threshold can be assumed for particulate matter [33]. Additional scientific evaluations confirm that health effects may occur even at low levels of exposure, particularly among children and other vulnerable populations [34].

Meteorological factors and seasonal variability also play an important role in modifying air pollution exposure and associated health outcomes. Temperature, atmospheric stability, wind patterns, and seasonal dynamics influence pollutant dispersion and secondary particle formation. Seasonal ozone peaks during warmer months and increased particulate matter accumulation during colder periods have been associated with higher rates of asthma exacerbations [19,22], highlighting the importance of integrating climatic variables into air-quality assessment and public health planning.

Evidence regarding sulfur dioxide (SO_2_) was limited and inconsistent across the included studies. While some analyses suggested weak or non-significant associations with asthma outcomes, the overall evidence was insufficient to establish a clear exposure–response relationship [8,9]. This inconsistency likely reflects reduced emissions in many regions, lower variability in exposure levels, and difficulties in separating SO_2_ effects from co-pollutants. Further research is required to clarify its independent role in pediatric asthma.

From a public health perspective, these findings reinforce the importance of continued efforts to reduce ambient air pollution exposure, particularly in urban environments where traffic-related emissions remain a dominant source. Although the included studies were observational and did not directly evaluate interventions, broader environmental health evidence suggests that reductions in air pollution are associated with improvements in lung development and respiratory outcomes in children [18]. Strengthening air-quality standards, improving urban planning, and promoting cleaner transportation systems may therefore contribute to reducing the burden of childhood asthma.

This review has several limitations. Heterogeneity in exposure assessment methods may introduce exposure misclassification, while variation in outcome definitions limits direct comparability across studies. Residual confounding from socioeconomic and environmental factors cannot be fully excluded. In addition, limited region-specific longitudinal evidence from Greece and restriction to English-language publications may have influenced the scope of included studies. Despite these limitations, the consistency of findings across populations and study designs strengthens confidence in the observed associations.

## 5. Conclusions

Ambient outdoor air pollution is a major and potentially modifiable environmental determinant of childhood asthma [4,8,12]. This 25-year systematic review (2000–2025) demonstrates consistent associations between exposure to PM_2.5_, PM_10_, and NO_2_ and both asthma incidence and exacerbation in children [1,2,3,10,16,18,25].

Adverse respiratory effects were documented even at concentrations below current regulatory limits [10,12,13]. The consistency of findings across cohort studies, time-series analyses, and multicenter European initiatives strengthens the evidence supporting a likely causal association [3,20,21,25,26,27].

Future research should focus on harmonized longitudinal exposure assessment, improved characterization of developmental windows, and integration of biological markers to clarify mechanistic pathways. Strengthening global surveillance and reducing ambient air pollution exposure remain essential public-health strategies to decrease the burden of childhood asthma worldwide [3]. These findings should be interpreted in the context of observational evidence and highlight priorities for future research and public-health policy.

## Figures and Tables

**Table 1:** Summary of selected representative epidemiological studies included in the review and examining the association between air pollution exposure and childhood asthma outcomes; full table provided in Supplementary Material.

Study	Country/Region	Study Design	Population	Pollutant(s)	Main Outcome
[8]	International	Systematic review/meta-analysis	Children	Traffic-related pollution, NO_2_	Increased risk of childhood asthma development
[9]	Global	Meta-analysis	Pediatric populations	Vehicle emissions	Higher asthma prevalence associated with traffic pollution
[10]	Multi-country	Cohort	Pediatric cohorts	PM_2.5_	Long-term PM_2.5_ exposure associated with increased asthma incidence
[11]	USA	Time-series	Pediatric asthma patients	PM_10_, O_3_	Short-term pollution associated with increased asthma symptoms
[18]	USA	Prospective cohort	Schoolchildren	PM_2.5_, NO_2_	Improved air quality associated with improved lung development
[19]	Greece	Time-series	Urban children	PM_10_	Increased pediatric hospital admissions during pollution peaks
[20]	Greece	Epidemiological study	Urban population	PM_2.5_, NO_2_	Urban air pollution associated with respiratory morbidity
[21]	Netherlands	Birth cohort	Children	PM_2.5_	Early-life exposure associated with reduced lung growth
[22]	Europe	Multicenter cohort	>300,000 children	NO_2_, PM_2.5_	Long-term exposure associated with increased asthma incidence

**Table 2:** Percentage Increase in Childhood Asthma Risk Associated with Major Air Pollutants.

Pollutant	Exposure Increase	% Increase in Asthma Risk	Key References
PM_2.5_	+10 μg/m^3^	20–30%	[8,10,20]
PM_10_	+10 μg/m^3^	15–25%	[11,15,28]
NO_2_	+10 μg/m^3^	15–30%	[8,12,21]
O_3_	High seasonal exposure	10–15%	[8,9]

**Table 3:** Summary of European and Greek Studies on Air Pollution and Childhood Asthma.

Study	Country/Region	Population	Pollutant	Main Outcome
[22]	Multi-country Europe	>300,000 children	NO_2_, PM_2.5_	↑ Asthma incidence
[21]	Netherlands	Birth cohort	PM_2.5_, NO_2_	↓ Lung growth
[26]	Spain	Schoolchildren	Traffic pollutants	↑ Asthma symptoms
[19]	Greece	Urban children	PM_10_	↑ Hospitalizations

## Data Availability

No new data were created.

## Supplementary Materials

The following supporting information can be downloaded at: https://www.mdpi.com/article/10.3390/arm94030031/s1, Table S1: Table 1. Overview of Studies on Air Pollution and Childhood Respiratory Health (1993–2024).
