# Supplementary material for: Association Between Air Pollution and Childhood Asthma: A Systematic Review of Recent Evidence
Source: Adv Respir Med. 2026 May 12;94(3):31. doi: 10.3390/arm94030031 (PMC13214496; doi:10.3390/arm94030031)
Supplement: Supplementary file 1 [file arm-94-00031-s001.zip › arm-4143048-supplementary.pdf]

Supplementary Material

Table S1: Table 1. Overview of Studies on Air Pollution and Childhood Respiratory Health (1993--2024).

Study (Author, Year)	Country/Region	Study Design	Population	Sample Size	Pollutant(s)	Exposure Type	Outcome	Key Findings
Khreis et al., 2017	International	Systematic review/meta-analysis	Children	—	NO_2, traffic-related pollution	Long-term	Incidence	↑ risk of asthma development
Gasana et al., 2021	Global	Meta-analysis	Children	—	Vehicle emissions	Long-term	Prevalence	↑ asthma prevalence
Wang et al., 2023	Multi-country	Cohort	Children	>100,000	$PM_{2.5}$	Long-term	Incidence	↑ asthma incidence
Delfino et al., 2014	USA	Time-series	Pediatric patients	—	PM_{10}, O_3	Short-term	Exacerbations	↑ symptoms and hospital visits
ECHO CREW, 2024	USA	Cohort	Prenatal/children	—	$PM_{2.5}$	Long-term	Incidence	↑ early-life asthma risk
Guarnieri & Balmes, 2014	Global	Narrative review	Children	—	Multiple pollutants	Both	Mechanisms	Oxidative stress & inflammation
Vrijheid et al., 2013	Europe	Cohort	Children	—	NO_2	Long-term	Incidence	↑ asthma risk
Gauderman et al., 2015	USA	Cohort	Schoolchildren	~2,000	$PM_{2.5}$, NO_2	Long-term	Lung development	Improved air → ↑ lung growth
Liu et al., 2022	Global	Time-series	Children	—	Mixed pollutants	Short-term	Exacerbations	↑ ER visits
Clark et al., 2010	Europe	Cohort	Children	—	$PM_{2.5}$	Long-term	Incidence	↑ asthma risk
Paciorek et al., 2020	USA	Cohort	Children	—	O_3	Short-term	Morbidity	↑ asthma exacerbations
Samoli et al., 2017	Greece	Time-series	Children	—	PM_{10}	Short-term	Hospitalizations	↑ pediatric admissions
Katsouyanni et al., 2020	Greece	Epidemiological	Urban population	—	$PM_{2.5}$, NO_2	Long-term	Morbidity	↑ respiratory outcomes
European Environment Agency, 2023	Europe	Report	General population	—	Multiple pollutants	Long-term	Exposure	Persistent elevated pollution levels

Brunekreef & Holgate, 2002	Global	Review	Children	—	Multiple pollutants	Both	Health effects	Strong association with respiratory disease
Dockery & Pope, 1993	USA	Cohort	Children	—	PM	Long-term	Respiratory outcomes	↑ respiratory symptoms
ISAAC II Study	Global/Greece	Cross-sectional	Schoolchildren	Large international	Urban pollutants	Long-term	Prevalence	↑ asthma prevalence
Hajat et al., 2021	USA	Epidemiological	Children	—	NO_2	Long-term	Exposure disparity	SES-related exposure differences
BREATHE Project, 2018	Spain	Cohort	Schoolchildren	~2,700	Traffic pollutants	Long-term	Symptoms	↑ asthma symptoms
PIAMA Study, 2015	Netherlands	Birth cohort	Children	~4,000	$PM_{2.5}$, NO_2	Long-term	Lung function	↓ lung growth
ESCAPE Project, 2014	Europe	Multicenter cohort	Children	>300,000	NO_2, $PM_{2.5}$	Long-term	Incidence	↑ asthma incidence
GA²LEN Study, 2016	Europe	Cohort	Children	—	NO_2	Long-term	Allergic disease	↑ asthma/allergy risk
Brugha & Grigg, 2014	UK	Review	Children	—	Urban pollution	Long-term	Respiratory outcomes	↑ infections/asthma
Clark et al., 2014	USA	Cross-sectional	Children	—	NO_2	Long-term	Exposure	Urban exposure inequalities
